# Bimanual Coordination and Right‐Hand Bias in Extractive Foraging by Wild *Sapajus libidinosus*

**DOI:** 10.1002/ajp.70165

**Published:** 2026-05-18

**Authors:** Valentina Truppa, Ilaria Soraci, Luca Antonio Marino, Dorothy M. Fragaszy, Patrícia Izar, Elisabetta Visalberghi

**Affiliations:** ^1^ Institute of Cognitive Sciences and Technologies, National Research Council (CNR) Rome Italy; ^2^ Department of Life Sciences and Systems Biology University of Torino Turin Italy; ^3^ Department of Sciences Roma Tre University Rome Italy; ^4^ Department of Psychology University of Georgia Athens Georgia USA; ^5^ Department of Experimental Psychology University of São Paulo São Paulo Brazil

**Keywords:** bimanual coordination, capuchin monkeys, handedness, laterality, manual dexterity, tube task

## Abstract

Bimanual coordination is considered a crucial factor in the evolution of manual lateralization. Experimental tasks requiring the simultaneous and coordinated use of both hands induce hand preferences at the population level across various nonhuman primate species studied in captive environments. However, to date, research investigating laterality in bimanual coordination within natural settings remains scarce, especially with respect to platyrrhine taxa. We assessed hand preference in a bimanual coordinated extractive foraging task by wild bearded capuchin monkeys, *Sapajus libidinosus*, living in the Cerrado/Caatinga ecotone, Northeast Brazil. Nineteen individuals were filmed during the consumption of four different nut species for 4 months. A detailed video‐coding analysis was conducted on their hand movements to extract the edible kernel(s) from the nuts. Overall, most individuals were lateralized, and as a group, capuchins significantly preferred their right‐hand fingers for extracting the kernel, thus suggesting an advantage of the left hemisphere for fine finger movements. The comparison with captive capuchins previously tested with an experimental bimanual coordinated task (the tube task), revealed that the direction of the preference did not differ between wild and captive individuals, although the latter tended to use their preferred hand more consistently. Our findings are in line with a growing body of evidence suggesting that bimanual coordination might have played a major role in the evolution of limb preference for motor action in primates and indicate that the study of coordinated bimanual behaviors in natural settings provides a valuable contribution to the understanding of manual laterality.

## Summary

- We assessed manual laterality in free‐ranging *Sapajus libidinosus* while foraging on four distinct nut species and compared their results with data from captive *Sapajus* spp. previously tested with the tube task.
- When wild capuchins used bimanual coordination, a significant preference for using their right‐hand fingers to extract the nut kernel emerged at the group level, implying a left‐hemisphere advantage for visually guided fine finger movements.
- Wild and captive individuals showed a similar right‐hand bias; however, the latter used their preferred hand more consistently, highlighting the importance of considering different settings and measures in the study of manual laterality.

## Graphical abstract

Wild *Sapajus libidinosus* exhibit a significant preference at the population level for using their right‐hand fingers to extract nut kernels when employing bimanual coordination.

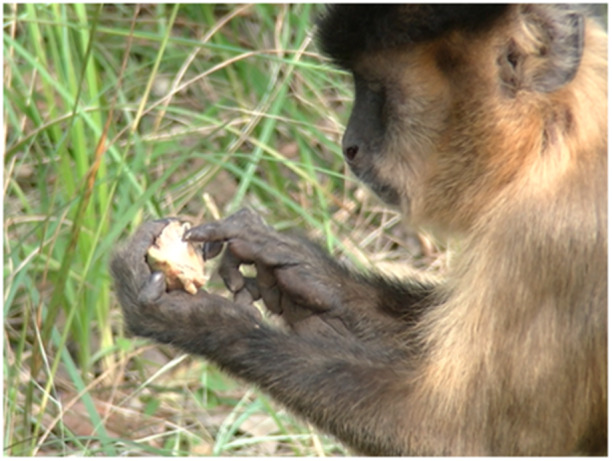

**Abbreviations**
ABS‐HI: Absolute handedness index
HI: Handedness index.

## 1 Introduction

Bilateral forelimb movements have expanded well beyond the bounds of locomotor behavior in primate evolution, as revealed by the extensive bimanual food processing and object manipulation typical of these species. Bimanual behavior refers to the simultaneous use of both hands to perform either differentiated or undifferentiated movements. In particular, we refer to bimanual coordination (Hopkins 1995) or role‐differentiated bimanual manipulation (Kimmerle et al. 2010) when the two hands act simultaneously as a synergy to achieve a specific goal with movements differentiated and coordinated. In this two‐handed behavior, one hand provides support, and the other hand performs finer movements (McGrew and Marchant 1997).

The ability to process different but parallel information during coordinated asymmetrical use of hands might be favored by the hemispheric specialization of manual functions, which is in line with the idea that lateralization increases neural capacity by avoiding duplication of functions in the two hemispheres (Levy 1977; Vallortigara and Rogers 2005). In accord with this hypothesis, asymmetrical bimanual behaviors have proven to be effective measures of population‐level hand preferences in human and nonhuman primates. Theoretical works and review articles have highlighted the importance of using bimanual tasks to assess hand preferences since the late 1980s, when the study of manual laterality in nonhuman primates received a new boost. MacNeilage et al. (1987), in their postural origins theory of handedness, proposed that primates initially developed a population‐level preference for the left hand (LH) for visually guided reaching. This was accompanied by a population‐level preference for the right hand (RH) for postural support. The demands for postural support from the hand decreased as terrestriality increased in some primate lineages (e.g., hominids), and as these lineages developed more advanced manipulative abilities, the specialization for postural support in the RH evolved into a population‐level RH preference for tasks that require fine, sequential manipulations and bimanual coordination. This theory has mostly not been supported as originally proposed, and later authors have proposed different trajectories for the evolution of manual asymmetry. Fagot and Vauclair (1991), in their critical review of the literature, highlighted the role of task demands, with only more complex tasks, such as bimanual coordination, capable of inducing an asymmetrical distribution of hand biases at the population level. Then, reviewing two decades of literature about manual laterality in nonhuman primates, Meguerditchian et al. (2013) addressed bimanual coordination as one of the main factors at play in inducing manual laterality and showed that tasks requiring bimanual coordination are more effective than unimanual tasks for finding a measurable hand bias in primates. In the last decade, interest in bimanual coordination has continued to grow, as demonstrated by several recent review and opinion articles. Prieur et al. (2019) highlighted how the comparative evidence about human and nonhuman animals' behavioral asymmetries helps to understand the processes that lead to strong human left‐hemisphere specialization. According to these authors, the manual laterality of modern humans is strengthened by the increasing level of complexity of our daily tasks, including bimanually coordinated actions and tool use. Finally, Nelson (2022) claimed that researchers examining nonhuman primates and young children have approached the topic of handedness from different viewpoints—evolutionary for primates and developmental for children. She highlighted the importance of measurement in the study of handedness and proposed that assessment methods may help guide future research into the origins of handedness. One promising behavior for facilitating multidisciplinary comparisons, she suggested, is role‐differentiated bimanual manipulation.

With respect to assessment methods, the experimental task that has been most commonly used to assess hand preference for bimanual coordination in nonhuman primates is the tube task originally proposed by William Hopkins to investigate manual laterality in chimpanzees (*Pan troglodytes*; Hopkins 1995). This task simulates natural extractive foraging activities and requires individuals to perform coordinated bimanual movements to retrieve food from a small tube (e.g., a cylinder or a pipe). Specifically, the ‘supporting’ hand stabilizes the tube, whereas the ‘dominant’ hand extracts the food by means of fine finger movements. The tube task is an effective and relatively simple testing protocol; therefore, over time, it has gained popularity as a standard measure of hand preference. Population‐level hand preferences emerged in many nonhuman primate species when this task was administered in captive or seminatural environments (e.g., chimpanzees: Hopkins et al. 2011; Llorente et al. 2011; Padrell et al. 2019; bonobos: Chapelain et al. 2011; Hopkins et al. 2011; gorillas, and orangutans: Hopkins et al. 2011; siamangs: Morino et al. 2017; Barbary macaques: Regaiolli et al. 2018; baboons: Vauclair et al. 2005; guenons and mangabeys: Maille et al. 2013; gracile capuchins: Meunier and Vauclair 2007; robust capuchins: Spinozzi et al. 1998; ring‐tailed lemurs: Regaiolli et al. 2016). It also appears to be a measure that is consistent over time: Padrell et al. (2019) reported that, in chimpanzees, the direction of hand preference (right vs. left) in the tube task remained stable after both short and long time periods. Moreover, Hopkins (2018) noticed that several investigators have observed digit use in different primate taxa when extracting food from the tube, and there is some evidence that single‐digit use induces an increased expression of strength and direction of handedness (chimpanzees: Hopkins 1995; siamangs: Morino et al. 2017; baboons: Vauclair et al. 2005; guenons and mangabeys: Maille et al. 2013; spider monkeys: Nelson and Boeving 2015). Therefore, he concluded, it is possible that variation in digit use may alter hand use and that this warrants further investigation.

Despite a growing body of studies investigating manual laterality for bimanually coordinated behavior in captive individuals, studies in wild populations (gorillas: Byrne and Byrne 1991; Salmi et al. 2016; Tamura and Akomo‐Okoue 2021; snub‐nosed monkeys: Zhao et al. 2010, 2012; bearded capuchins: Salmi et al. 2023) and semi‐wild populations (chimpanzees: Forrester et al. 2016) are still scarce. At present, very little research has been conducted in this respect, especially in platyrrhine taxa, as a consequence of the fact that field studies on manual functions can be particularly challenging in small‐sized arboreal monkeys such as platyrrhines.

Among platyrrhines, capuchin monkeys (genera *Cebus* and *Sapajus*) have received much attention in terms of manual functions, including laterality (Nelson et al. 2025), due to their high level of manual dexterity, which distinguishes them from other neotropical primates (Truppa et al. 2019a). Some studies on hand preference have investigated the effect of bimanual coordination using the tube task in captive settings. Among those samples that showed a significant bias, most preferred the RH for dexterous action (*Sapajus* spp.: Spinozzi et al. 1998; *S. apella*: Phillips and Hopkins 2007; *S. robustus* and *S. flavius*: De Andrade and De Sousa 2018; but see De Andrade and De Sousa 2018 for opposite results in *S. libidinosus*). More recently, Salmi et al. (2023) conducted a field experiment and reported a group‐level hand preference for coordinated bimanual behavior in wild *S. libidinosus*. When feeding on crabs, these monkeys hold a crab with their LH while the RH is used to pull off parts of the crab. The authors claimed that their study was limited by a small and unequal sample size (bimanual task: *N* = 11, 9 M and 2 F), which was a consequence of conducting the study on a wild group with voluntary subject participation. Indeed, field studies can be very challenging. Again, it seems crucial to conduct further studies on natural primate populations for activities that are comparable to the tasks used in captive studies and that involve several individuals for an adequate number of trials.

In this respect, an interesting case is that of free‐ranging bearded capuchins (*Sapajus libidinosus*), which live in Fazenda Boa Vista (FBV), Piauí, Brazil. These monkeys regularly use stones as tools to crack open hard‐shelled nuts (Spagnoletti et al. 2011). Their actions while cracking nuts have been studied extensively to understand how they use stones as tools (Fragaszy et al. 2020, 2023). However, the manner in which they extract kernels has not yet been studied in detail, neither to describe hand movements nor to evaluate the laterality pattern associated with this behavior.

Our preliminary observations indicated that kernel extraction indeed resembles the extraction process required by the tube task conceived by Hopkins (1995) to study bimanual coordination in captive nonhuman primates. Specifically, it encompasses bimanually coordinated movements in which one hand moves and stabilizes the nut, whereas the fingers of the other hand extract the kernel or pieces of it.

The main aim of this study was to determine whether wild bearded capuchin monkeys exhibit significant preferences at either the individual or group level for bimanual coordination involving precise finger movements. For this purpose, we examined how *Sapajus libidinosus* of FBV used their hands to extract the kernel from hard nuts, with special attention given to coordinated motor patterns between the two hands. We also assessed sex‐ or age‐related differences. In line with the task‐complexity hypothesis (Fagot and Vauclair 1991) and consistent with the majority of data on capuchins for bimanually coordinated behavior, we expected to find a group‐level hand preference. Moreover, we evaluated whether the laterality pattern varies between wild and captive capuchins tested with the tube task (Spinozzi et al. 1998).

## 2 Methods

### 2.1 Ethics Statement

This research was approved by IBAMA SISBIO (28689‐3 and 28689‐8) and CNPq (002547/2011‐2 and 000511/2015). Moreover, it adhered to the American Society of Primatologists (ASP) Principles for the Ethical Treatment of Non‐Human Primates and followed the American Society of Primatologists Code of Best Practices for Field Primatology. Finally, it adhered to the legal requirements of Brazil, where the research was conducted.

### 2.2 Site

The study site, Fazenda Boa Vista (hereafter FBV), is a privately owned area of approximately 13 km² situated 21 km northwest of the town of Gilbués (9°39′36″ S, 45°25′10″ W) in the northeastern Brazilian state of Piauí. FBV is in the ecotone between the *Cerrado* (open woodland) and *Caatinga* (semiarid) biomes (Oliveira and Marquis 2002) and occupies an area where capuchin monkeys are under little anthropogenic disturbance (Spagnoletti et al. 2017). This site is located on a plain approximately 420 m above sea level and is characterized by low‐nutrient sandy soils, a seasonal climate with a dry season from April to September, a rainy season from October to March, and interannually variable precipitation (800–1600 mm; Oliveira and Marquis 2002). Spagnoletti et al. (2011) reported that embedded fruits exploited by capuchins in FBV include several species of palm nuts (e.g., piassava, *Orbygnia* spp. and tucùm, *Astrocaryum campestre*) and other encased food items (e.g., fruta‐danta, family Icacinaceae; caju, family Anacardiaceae; caroba, family Bignoniaceae; manioca‐brava, family Euphorbiaceae).

Importantly, in FBV the vegetation allows good visibility of the monkeys and capuchins spend about 30% of their active time on the ground (Wright et al. 2019); therefore, it is an ideal site to film monkeys' manual behaviors (Truppa et al. 2019b).

### 2.3 Study Group

Nineteen wild bearded capuchin monkeys (*Sapajus libidinosus*) of both sexes and different ages were filmed while extracting kernel from nuts (see Table 1). For 18 out of 19 monkeys, we managed to collect a sufficient number of videos to conduct reliable statistical analyses on laterality in bimanual coordination (see Section 2.5 below). The monkeys live in one group of 21–23 individuals, depending on the different periods of data collection. In FBV, these monkeys rely on the consumption of food protected by hard matrices, such as hard nuts, for a good part of their diet (Izar et al. 2022). All monkeys were habituated to human observers at close range and were individually identified.

**Table 1:** Sex, age, class of age, number of nut species manipulated, number of independent extracting bouts, and individual manual laterality scores for coordinated bimanual and hand‐mouth behaviors of capuchins in the study group.

Subjects	Age (months)	Class of age	Nut species	(a) Bimanual coordination (preference for extracting)						(b) One hand‐mouth coordination					
				Total	RH	LH	HI	*z*	cutoff	Total	RH	LH	HI	*z*	cutoff
Males															
Mansinho	180^a^	A	3	67	40	27	0.2	Np	R	30	19	11	0.3	Np	R
Jatobá	168^a^	A	4	123	63	60	0.0	Np	Np	38	19	19	0.0	Np	Np
Teimoso	168^a^	A	3	92	82	10	0.8	R***	R	31	9	22	−0.4	L**	L
Tomate	89	A	3	53	46	7	0.7	R***	R	32	9	23	−0.4	L**	L
Catu	87	A	3	70	8	62	−0.8	L***	L	46	35	11	0.5	R***	R
Coco	58	I	3	100	66	34	0.3	R**	R	47	12	35	−0.5	L***	L
Presente	36	I	4	125	110	15	0.8	R***	R	11	2	9	—	—	—
Cachaça	26	I	4	109	75	34	0.4	R***	R	45	13	32	−0.4	L**	L
Females															
Piaçava	180^a^	A	3	76	7	69	−0.8	L***	L	43	14	29	−0.3	L**	L
Chuchu	156^a^	A	4	89	82	7	0.8	R***	R	39	17	22	−0.1	Np	Np
Dita	132^a^	A	4	115	112	3	0.9	R***	R	82	32	50	−0.2	L*	L
Doree	78	A	4	101	70	31	0.4	R***	R	16	5	11	—	—	—
Pamonha	64	A	3	82	46	36	0.1	Np	Np	22	13	9	—	—	—
Paҫoca	64	A	4	117	104	13	0.8	R***	R	6	1	5	—	—	—
Chani	39	I	2	14	2	12	—	—	—	0	0	0	—	—	—
Divina	18	I	3	45	45	0	1.0	R***	R	44	14	30	−0.4	L**	L
Donzela	16	I	3	64	10	54	−0.7	L***	L	18	17	1	—	—	—
Patrícia	16	I	4	34	30	4	0.8	R***	R	36	21	15	0.2	Np	R
Titia	16	I	3	70	68	2	0.9	R***	R	11	3	8	—	—	—

*Note:* A, adult (≥ 5 years); cutoff, preference according to cut‐off points HI ≥ +0.20 and HI ≤ −0.20 (Hopkins 2013a).

Abbreviations: HI, handedness index; I, immature (< 5 years); L, left handed; LH, left hand; Nut species, number of different nut species manipulated; Np, Nonpreferent; R, right handed; RH, right hand; *z*, preference according to binomial *z* scores (Binomial test).

*p < 0.05;

** *p* < 0.01;

*** *p* < 0.001.

^a^ Estimated age on the basis of body mass and behavior when they were first encountered by the Etho*Cebus* team (age referred to when data collection began).

### 2.4 Data Collection

Three observers (L. A. M., M. J. F. O., V. T.) filmed the monkeys for a total of 4 months: 3 months in the dry season of 2014 (June, L. A. M., V. T.; July‐August, L. A. M., M. J. F. O.) and 1 month at the beginning of the rainy season of 2015 (November, M. J. F. O., V. T.). The capuchins were observed throughout their daily activities from 6:30 to 16:30, 5 days per week. Data collection employed a high‐speed full HD digital camcorder (JVC GC‐PX100) equipped with a 10× zoom lens alongside a digital reflex camera (Canon EOS 600D Full HD) fitted with an EF‐S 55–250 mm f/4–5.6 IS zoom lens. Video recordings were captured at a resolution of 1080p and a frame rate of 60 fps in MOV format, providing an adequate number of frames for detailed coding. Recordings were performed at distances ranging from 2 to 5 meters from the subjects. The monkeys appeared unperturbed by the observer's proximity within this range.

**Figure 1:**
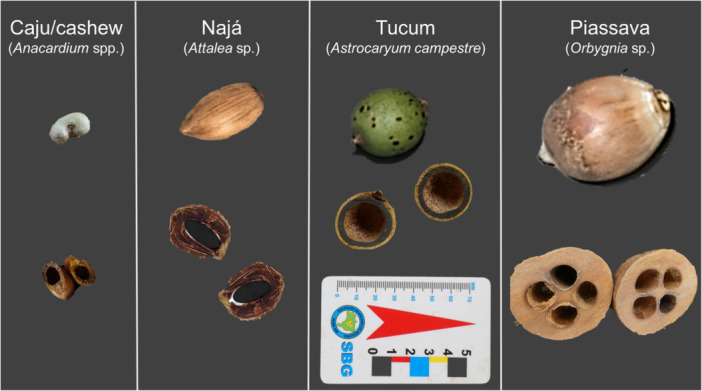
The four different species of nuts consumed by bearded capuchins in Fazenda Boa Vista, on which data collection was conducted. The cashew nut is the fruit of the cashew tree, whereas the other nuts are the fruits of palm trees.

Before we started filming the capuchins, we collected four different species of nuts in their home range: caju/cashew (*Anacardium* spp.), najá (*Attalea* sp.), tucum (*Astrocaryum campestre*), and piassava nuts (*Orbygnia* sp.), shown in Figure 1. In FBV, the nuts of the piassava, the tucum, and the najá palm trees are mainly available during the dry season and are relatively easy to collect because these palms produce fruits at the ground level. Cashew nuts are available only at the end of the dry season (green nuts) and at the beginning of the rainy season (dry nuts). The capuchins of FBV extract the kernel from both green and dry cashew nuts (Visalberghi et al. 2016).

To film as many individuals as possible engaged in kernel extraction, we provided the monkeys with nuts in an opportunistic way whenever a tool‐user monkey approached an anvil where at least one tool—typically a stone—was available. We also provided cracked piassava, tucum, and najá nuts to young capuchins up to 18 months of age because they were not yet capable of cracking nuts by using tools. The same was done with an adult female (Pamonha) who has not been observed to crack piassava nuts using tools. Handling pieces of nuts cracked open by other individuals reflects the monkeys' normal experience. In fact, years before becoming proficient tool users, young capuchins of FBV scrounge pieces of nuts left by tool‐using group members. Scrounging involves manipulating broken nuts and extracting nut kernels from them (Resende et al. 2021). Cashew nuts are an exception in this sense, as sometimes capuchins of FBV can open these nuts by rubbing them against the bark of the trees (green cashew nuts) or biting them with their teeth (dry cashew nuts), thus making the use of tools optional (Visalberghi et al. 2021). Therefore, cashew nuts were always provided unbroken to all the individuals of our study group.

### 2.5 Data Coding

The videos were initially viewed to select those that allowed us to clearly distinguish the monkeys and the behaviors of interest for coding. The videos selected were subsequently coded through detailed analysis via Behavioral Observation Research Interactive Software (BORIS) version 7.1.3 (Friard and Gamba 2016). The slow‐motion and zoom functionalities of this software enabled a precise analysis of the capuchins' hand movements during extraction. All videos were coded by one observer, and 20% of the videos were coded by another observer to assess interobserver reliability (Cohen's kappa = 0.93).

For each individual, for each video on each nut, we coded whether the manipulation to extract the kernel occurred on the ground or in a tree. A description of individual behaviors is reported in Table 2 (see also Supplementary Video S1). Extracting behaviors by each hand were categorized as bimanual or unimanual, and the coordination of the hands with other parts of the body were coded (categories were two hands‐mouth coordination, one hand‐mouth coordination, bimanual coordination, and two hands‐mouth‐foot coordination). Hand use was recorded as “events” and “bouts” (Hopkins 2013a). For events, we considered every single instance, while bouts were defined as a sequence of identical instances that occurred immediately after the nut had been transferred from one hand to the other or taken from a substrate (usually soil, anvil, or branch) or if it was preceded by a different manipulation action. This was especially important for the evaluation of manual preference because it is possible that the choice of a hand is influenced by the fact that the hands are already acting according to a precise motor pattern (Byrne and Byrne 1991). For example, in the case of extracting nut kernels, if a monkey performs an extraction action using one hand to hold the nut and the other hand to extract the kernel, it is possible that it will perform a series of actions adopting the same motor pattern. In other words, in a series of identical actions close together in time in which the motor scheme is always the same, it is possible that the actions that follow the first action are not independent of the choice made to perform the first action. For this reason, we preferred to use isolated events and bouts, rather than the total number of events, as the unit of analysis.

**Table 2:** Manipulative patterns within each behavioral category analyzed in relation to nut consumption by bearded capuchins in Fazenda Boa Vista.

Behavior	Description
Extracting	
Two Hands‐Mouth coordination	Grasping a nut with both hands simultaneously and bringing it to the mouth to extract the kernel using the canine teeth (Figure 2b).
One Hand‐Mouth coordination	Grasping a piece of nut with one hand and bringing it to the mouth to extract the kernel using the canine teeth (Figure 2b).
Bimanual coordination	Grasping a piece of nut with one hand while using the distal phalanges of one or more fingers of the other hand to extract the kernel (Figure 2b).
Two Hands‐Mouth‐Foot coordination	Grasping a piece of nut with both hands and one foot simultaneously and bringing it to the mouth to extract the kernel using the canine teeth (Figure 2b).
Rolling	Rolling the nut ‐ or fragments of it ‐ using one or both hands with the fingers extended against a rough surface (e.g., tree bark) or between the palms of the hands or between the palm of one hand and the side of the other hand/forearm (Figure 3a).
Beating	Beating the nut on a surface with one or both hands (Figure 3b).
Table (Hill 1960)	Recovering pieces of food from the forearms aligned and in contact with each other with the volar surface facing upwards to form a sort of “table”. This behavior prevents the extracted pieces of nut from falling. The arms are raised slightly to an angle that prevents the crumbs from falling, at which point the animal bends its head forward to reach the pieces of food with its mouth (Figure 3c).
Foot grasping	Grasping and holding the nut with one foot (Figure 3d).
Tearing off	Tearing off a piece of the nut shell with one hand or mouth (Figure 3e).
Rubbing	Grasping the nut firmly with one or both hands and moving it back and forth over a surface (e.g., tree bark) while applying pressure (Figure 3f).
Tapping	Hitting gently and repeatedly the nut with the tip of one or more fingers of one hand while holding it steady with the other hand or resting it on a surface (Figure 3g).

In addition to the extraction behavior, we coded the following manipulation behaviors performed during the processing of nuts: tapping, rolling, rubbing, table, tearing off, nut beating, and foot grasping (the behaviors are described in Table 2) and shown in Supplementary Video S2. The hand/foot preference (left or right) and/or the use of the mouth were coded for all the above behaviors, apart from “table”, which always involves the simultaneous and undifferentiated use of both forearms.

### 2.6 Data Analysis

To analyze hand preferences at the individual level, four scores were calculated:

(1) The HI (Handedness Index) score, which establishes the hand preference direction on the basis of the following formula: HI = (R–L)/(R + L), where R represents the frequency of use of the RH and L the frequency of use of the LH. The resulting values allowed the characterization of each individual as falling along a continuum that goes from an extreme negative value equal to −1.0 (exclusive use of the LH) to an extreme positive value equal to +1.0 (exclusive use of the RH);

(4) The ABS‐HI (Absolute Handedness Index) score, which establishes hand preference strength, regardless of whether an individual is right or left handed. The ABS‐HI represents the absolute value of the handedness index HI. Since these are absolute values, the range of variation of the ABS‐HI scores ranges from 0.0 (use of the two hands with equal frequency) to 1.0 (exclusive use of one hand);

1. The binomial *z* score (Siegel and Castellan 1988), whose values ≥ 1.96 indicate an RH preference, and those ≤ −1.96 indicate an LH preference (*p* < 0.05). With a *z* score between these two values, an individual is classified as ambidextrous;
2. The HI cutoff points are +0.20 and −0.20, whereas all others are classified as nonpreferent. This second classification follows the criterion suggested by Hopkins (2013a), who reported that HI values of +0.20 and −0.20 roughly correspond to *z* scores of ±1.96 when a minimum of 30 responses are obtained to measure hand preference. However, the advantage of adopting this approach is that HI scores are not sensitive to variations in sample size.

A Chi^2^ goodness‐of‐fit test was used to determine if the number of lateralized and non‐lateralized individuals was equally distributed. Analogously, the same test was used for the subsample of lateralized individuals to determine if the number of right‐handers and left‐handers was equally distributed. The effect size was evaluated with Phi (*Φ*).

Before proceeding to the other group‐level analyses, preliminary Kolmogorov‐Smirnov tests were applied to the data distributions to assess whether they significantly deviated from normality. Depending on the results of the normality tests, we then proceeded with parametric or nonparametric statistical tests for the subsequent analyses. In particular, the following tests were used:

1. The one‐sample Wilcoxon signed‐rank test to evaluate whether the median HI values of the sample for bimanual coordinated behavior differed from a chance distribution with a median of 0 (Z statistic divided by the square root of the sample size, i.e., r, was used to calculate the effect size);
2. The one‐sample *t*‐test to evaluate whether the mean HI values of the sample for the hand‐mouth coordinated behavior differed from a chance distribution with a mean of 0 (Cohen's D was used to calculate the effect size);
3. The Mann–Whitney *U* test to assess differences in the direction (HI) and strength (ABS‐HI) of hand preference in relation to the age class and sex of individuals for bimanual coordinated behavior and to compare the direction (HI) and strength (ABS‐HI) of hand preference of wild and captive capuchins for bimanual coordinated behavior (rank‐biserial correlation coefficient, i.e. r_b_, was used to calculate the effect size);
4. The analysis of variance (ANOVA) to assess differences in the direction (HI) and strength (ABS‐HI) of hand preference in relation to the age class and sex of the individuals for coordinated hand‐mouth behavior (partial Eta squared, i.e., *η*_*p*_^*2*^, was used to calculate the effect size).

Statistical significance was set at a confidence level of 95% (*p* ≤ 0.05). All tests were two‐tailed.

## 3 Results

### 3.1 Manipulative Behaviors Observed to Extract the Kernel From Nuts

**Figure 2:**
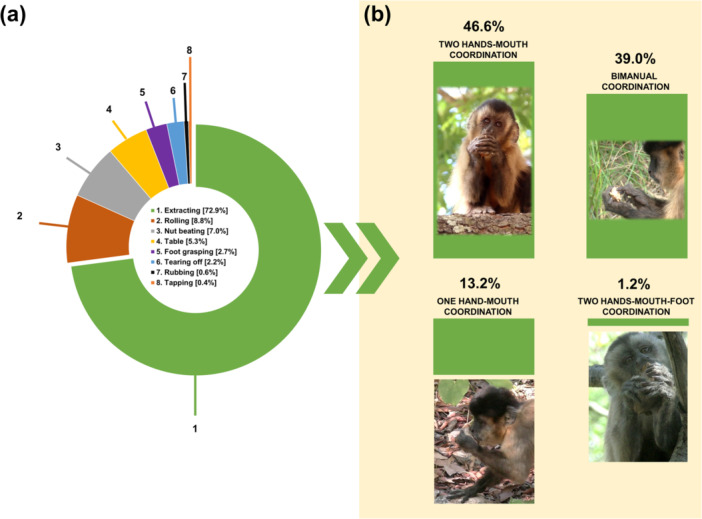
Percentages of use of manipulative behaviors considered in this study (a) and details on different techniques of extraction behavior (b).

When capuchin monkeys manipulated nuts to extract the kernel, they were on the ground 60.6% of the time. Overall, extraction behavior bouts accounted for 72.9% of the total nut handling bouts used by bearded capuchins in our study group (Figure 2a). The monkeys used bimanually coordinated behaviors during 39.0% of the extraction bouts to remove the kernel from the shell (Figure 2b), somewhat less often than they used both hands simultaneously in coordination with the mouth (46.6% of bouts). A low percentage of extraction bouts were performed using only one hand in coordination with the mouth (13.2%) or both hands in coordination with the mouth and one foot (1.2%).

**Figure 3:**
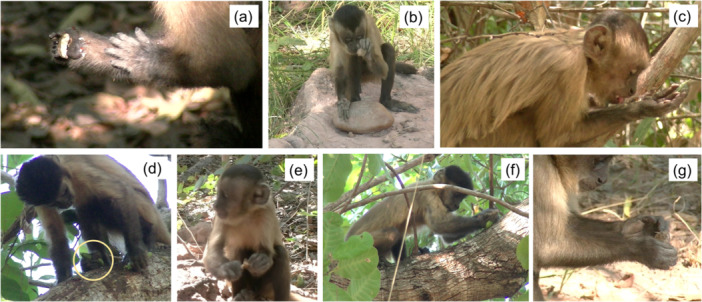
Images depicting nut manipulation behaviors other than the extraction techniques: Rolling (a), beating (b), table (c), foot grasping (d), tearing off (e), rubbing (f) and tapping (g). Descriptions of these behaviors are provided in Table 2.

Taken together, the other behaviors considered (tapping, rolling, rubbing, table, tearing off, nut beating, and foot grasping) accounted for approximately one‐third (27.1%) of the total manipulation bouts (Figure 2a and Figure 3).

### 3.2 Manual Laterality

#### 3.2.1 Bimanual Coordination and Hand‐Mouth Coordination

Table 1 reports individual data on the hand preferences of 19 bearded capuchins (8 males, 11 females) for (a) differentiated bimanually coordinated bouts and (b) hand‐mouth extraction bouts where only one hand was used in coordination with the mouth. These were the only two extraction behaviors for which it was possible to establish hand preferences.

For each of these two behaviors, HI scores and hand preferences at the individual level were calculated only for those monkeys for which at least 30 extraction bouts were available: 18 individuals for bimanual coordination and 12 individuals for hand‐mouth coordination.

On the basis of binomial *z* scores, most individuals in our study group were lateralized for bimanual coordination [15 out of 18 individuals, *Chi* ^*2*^ = 8, *df* = 1, *p* = 0.005, *Φ* = 0.58] but not for hand‐mouth coordination [8 out of 12 individuals, *Chi* ^*2*^ = 1.3, *df* = 1, *p* = 0.248, *Φ* = 0.29]. A significantly greater number of lateralized individuals appear to prefer the RH [12 out of 15 individuals, *Chi* ^*2*^ = 5.4, *df* = 1, *p* = 0.020, *Φ* = 0.60] in bimanual coordination, whereas an opposite trend was found in hand‐mouth coordination [7 left‐handers out of 8 lateralized individuals, *Chi* ^*2*^ = 4.5, *df* = 1, *p* = 0.034, *Φ* = 0.75].

**Figure 4:**
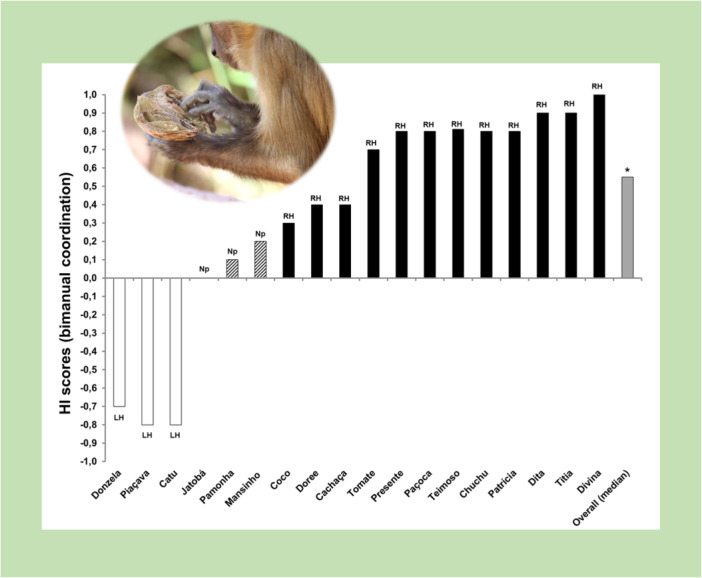
Individual Handedness Index scores for bimanual coordinated bouts and the median Handedness Index score for the group.

In addition, bearded capuchins showed a significant preference at the group level for using their RH to extract the nut kernel in bimanually coordinated behavior [*N* = 18, median HI = 0.55, one‐sample Wilcoxon signed‐rank test, *W* = 124.5, *p* = 0.022, *r* = 0.54; Figure 4]. In contrast, no hand preference emerged at the group level when they used one hand in coordination with the mouth [mean HI = −0.14, *SE* = 0.09, one‐sample *t*‐test, *t*(11) = −1.5, *p* = 0.160, Cohen's D = −0.435].

No significant differences were found regarding the direction (HI) and strength (ABS‐HI) of hand preference in relation to the age class (11 adults vs. 7 immatures) or sex (8 males vs. 10 females) of the individuals for bimanual coordination (see Table 3). Similar results were obtained concerning the direction (HI) and strength (ABS‐HI) of hand preference in relation to the age class (8 adults vs. 4 immatures) or sex (7 males vs. 5 females) of the individuals for one hand‐mouth coordination (see Table 4).

**Table 3:** Results of Mann–Whitney *U* tests on the direction (HI) and strength (ABS‐HI) of hand preference in relation to the age class or sex of the individuals for bimanual coordination.

	Sex					Age class				
	Median males	Median females	U	*p*	r_b_	Median adults	Median immatures	U	*p*	r_b_
HI	0.35	0.80	27.0	0.242	−0.27	0.40	0.80	26.0	0.252	−0.27
ABS‐HI	0.55	0.80	21.0	0.081	−0.41	0.80	0.80	29.5	0.400	−0.20

Abbreviations: ABS‐HI, Absolute Handedness Index; HI, Handedness Index.

**Table 4:** Results of ANOVAs on the direction (HI) and strength (ABS‐HI) of hand preference in relation to the age class or sex of the individuals for actions coordinating one hand with the mouth.

	Sex					Class of age					Sex X Class of age		
	Mean males	Mean females	F (d.f.)	*p*	*η*_*p*_^*2*^	Mean adults	Mean immatures	F (d.f.)	*p*	*η*_*p*_^*2*^	F (d.f.)	*p*	*η*_*p*_^*2*^
HI	−0.13	−0.16	0.14 (1,8)	0.722	0.017	−0.07	−0.27	0.74 (1,8)	0.415	0.085	1.82 (1,8)	0.214	0.186
ABS‐HI	0.36	0.24	1.97 (1,8)	0.198	0.198	0.27	0.37	1.43 (1,8)	0.266	0.152	0.02 (1,8)	0.880	0.003

Abbreviations: ABS‐HI, Absolute Handedness Index; d.f., degrees of freedom; HI, Handedness Index.

#### 3.2.2 Comparison Between Wild and Captive Capuchins

The hand preference of wild capuchin monkeys in our study group was compared with that of captive robust capuchins (*Sapajus* spp.) previously tested at the Primate Center of the Italian National Research Council with the tube task (a hanging vertical transparent tube with a 1.5‐cm hole made in the side) by Spinozzi et al. (1998). In particular, the HI values for the tube task in crouched posture were used as comparison data (Spinozzi et al. 1998, p. 187, Table 2, crouched condition). Although Spinozzi et al. (1998) found that the monkeys' posture did not affect either the direction or strength of hand preference in the tube task, we used data from crouched/seated postures for comparison because they paralleled the posture we typically observed in wild capuchins while they extracted nut kernels.

**Figure 5:**
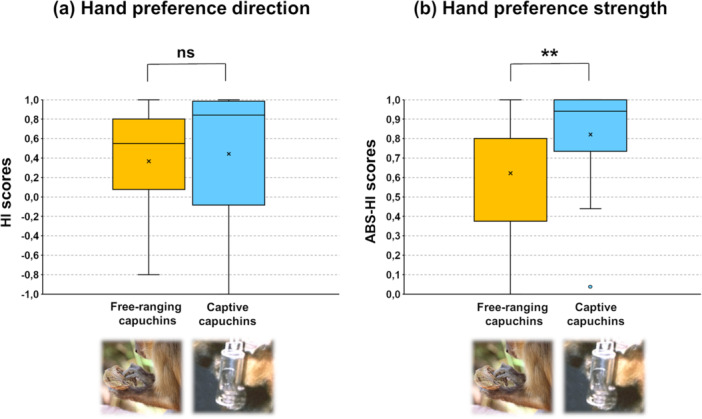
Comparison between laterality indices for bimanual coordination obtained in this study on nut consumption by capuchins in Fazenda Boa Vista and those obtained with the tube task by captive capuchins (Spinozzi et al. 1998). No significant difference emerged regarding hand preference direction (Handedness Index, panel (a), whereas wild capuchins showed a significantly lower hand preference strength than captive individuals (Absolute Handedness Index, panel (b). Whiskers represent 1.5 × IQR (Interquartile range).

Regarding the hand preference direction (HI), both wild (see Section 3.2 above) and captive capuchins (Spinozzi et al. 1998, p. 185) showed a significant preference for using their RH for extracting the kernel during coordinated bimanual bouts. No significant difference emerged when the median HI scores of the two samples were compared [N_wild_ = 18, median HI_wild_ = 0.55; N_captive_ = 26, median HI_captive_ = 0.84, Mann–Whitney *U* test, *U* = 175.0, *p* = 0.158, r_b_ = −0.21; Figure 5a]. However, wild capuchins showed a significantly lower hand preference strength (ABS‐HI) during bimanual coordination than captive individuals did [N_wild_ = 18, median ABSHI_wild_ = 0.80; N_captive_ = 26, median ABSHI_captive_ = 0.94, Mann–Whitney *U* test, *U* = 124.5, *p* = 0.009, r_b_ = −0.40; Figure 5b].

## 4 Discussion

This study analyzed the manipulation patterns and manual preferences during nut consumption by wild bearded capuchin monkeys in Northeast Brazil. The behaviors observed concerned the manipulation of four different species of nuts present in their natural environment. In particular, we described the behaviors exhibited by capuchin monkeys to obtain the kernels once the nuts had been cracked open. We found a significant RH preference at the group level for the action of extracting the kernel during bimanually coordinated behavior. The comparison with captive capuchins previously tested at CNR (Italy) with the tube task, a task eliciting bimanual coordination, revealed that the direction of the preference did not differ between wild and captive individuals, although the latter tended to use their preferred hand more frequently.

### 4.1 Manipulative Behaviors Observed to Extract the Kernel From Nuts

The different manipulation patterns used by capuchin monkeys to extract the kernel from nuts reflect the high manual dexterity of the species (Fragaszy et al. 2004; Truppa et al. 2019a). Capuchins constantly alternated between coordinating their two hands with each other (greater precision) and coordinating their hand(s) with their mouth (greater strength) because extraction requires precise yet energetic movements. In fact, whereas bimanual coordination had the advantage of allowing visual guidance and therefore greater precision of hand movements, the use of the hand(s) and mouth, and to a lesser extent, one of the feet, allowing greater force to be exerted.

The extraction actions were accompanied by other manipulative behaviors, such as *rolling* and *tearing off*, which facilitated the process of separating the edible part of the kernel from the outer shell and other inedible parts of the nut. These behaviors are part of a diversified repertoire that wild capuchin monkeys employ during their foraging activities, which appears early in their development (Araujo et al. 2024). The exploitation of hard‐to‐obtain resources requires sustained effort and skill, as well as the ability to use various parts of the body in a coordinated manner, sometimes with the help of objects and substrates found in the environment (Fragaszy et al. 2004; Truppa et al. 2019a). Moreover, as previously observed in the consumption of other resources, such as underground storage organs (Truppa et al. 2019b), bearded capuchins show flexibility in performing certain behaviors. For example, rolling can be performed by rolling food between the palms of both hands, or by rolling food between a substrate (e.g., tree bark) and the hands (or a single hand). In capuchins, the ability to coordinate various parts of the body with each other and with objects/substrates is supported by movement skills, physical strength, and advanced visuospatial abilities.

### 4.2 Manual Laterality

#### 4.2.1 Bimanual Coordination and Hand‐Mouth Coordination

The frequency of use of the hand used to perform the most complex bimanually coordinated action (extracting the edible part with fine finger movements; see Supplementary Video S3) was assessed both at the individual level and at the group level. The latter, in particular, allows conclusions to be drawn at the population level and, therefore, suggests the presence of forms of hemispheric specialization for the species. The results revealed that almost all individuals, regardless of sex and class of age, had a significant manual preference. Interestingly, all four individuals between eighteen and sixteen months showed strong preferences typical of adults. At the group level, there was a significant preference for using the RH during the bimanual extraction of nut kernels, suggesting an advantage of the left hemisphere for precise finger movements. This leads to a mirrored preference for the LH to hold and orient the nut in space to facilitate extraction by the RH, suggesting an advantage of the right hemisphere for spatial information in visually guided actions. In contrast, no hand preference emerged at the group level when capuchins used one hand to hold and orient the nut in space to facilitate extraction by the canine teeth (one hand‐mouth coordination); a result consistent with previous observations in capuchins of FBV, wherein they used one hand in coordination with their mouth during the consumption of tubers (Whishaw et al. 2024).

Our results represent one of the first reports of manual laterality for a natural task requiring bimanual coordination in capuchin monkeys, and, more generally, in platyrrhine monkeys. They are consistent with the suggestions of several authors, who, after reviewing numerous studies on manual laterality in nonhuman primates, have suggested that bimanual coordination is one of the factors that contributes the most to functional asymmetries in the use of hands (e.g., Meguerditchian et al. 2013; Nelson 2022; Prieur et al. 2019). Our findings add to the still scarce evidence of laterality in bimanual behavior in wild nonhuman primates (e.g., gorillas: Salmi et al. 2016; Tamura and Akomo‐Okoue 2021; snub‐nosed monkeys: Zhao et al. 2010, 2012; capuchin monkeys: Salmi et al. 2023) and to the more abundant evidence that has accumulated over the past three decades on captive individuals (for a review, see Meguerditchian et al. 2013). In particular, our study corroborates the results recently reported by Salmi et al. (2023) in another population of wild bearded capuchin monkeys of Northeast Brazil inhabiting a mangrove forest for a different natural foraging activity. These capuchins presented a pattern of laterality in bimanual coordinated behavior of crab pulling, i.e., dismembering the crabs they fed on with one hand (typically the right), keeping the crab in place while the other (typically the left) pulled off parts of the crab.

Overall, these studies support the task‐complexity hypothesis proposed by Fagot and Vauclair (1991) to explain the relationship between individual‐ and group‐level manual lateralization and task complexity in nonhuman primates. According to this model, tasks involving particular cognitive abilities, such as visuospatial control of a cursor on a computer monitor to collide with a target stimulus, and complex motor actions, such as bimanual coordination, can elicit preferences at both the individual and species levels and therefore can highlight underlying hemispheric specializations.

Unfortunately, the picture is less clear in regard to the direction of manual preference found in different species or in different studies or tasks on the same species. MacNeilage et al. (1987), in their theory of the postural origins of manual dominance, suggested that in arboreal species the RH is not preferred for manipulative actions but is mainly specialized for postural support; instead, the RH becomes the dominant hand in bimanual coordination tasks in terrestrial primates and is no longer subject to the biomechanical constraints of an arboreal life. Our results are not consistent with this prediction, as capuchins, despite being arboreal primates, prefer to use their RH for complex tasks requiring bimanual coordination and fine manipulation. More generally, it is difficult to interpret the overall heterogeneous picture of the results across capuchins' studies within this theoretical framework (see Salmi et al. 2023 for a review). In relation to the theory of the postural origins of manual dominance, it is necessary to stress that although *Sapajus* includes species classified as arboreal, the capuchins of FBV used terrestrial substrates more than a quarter of the time (27%; see Wright et al. 2019). It has been suggested that terrestrial habits increase the likelihood of discovering solutions to foraging problems through tool use (e.g., Visalberghi et al. 2005), and that terrestriality appears to promote orthograde (bipedal) behaviors associated with tool use (Wright et al. 2019). Further investigation is required to determine whether a more terrestrial lifestyle influences forelimb coordination in a manner consistent with the framework proposed by MacNeilage et al. (1987). Additional data from wild populations are necessary to clarify this relationship.

#### 4.2.2 Comparison Between Wild and Captive Capuchins

The data collected on wild capuchins for this study were compared with those of a previous study conducted by Spinozzi et al. (1998) on a sample of captive capuchins tested with the analogous tube task. We found that the two samples did not differ in terms of hand preference direction; however, a difference emerged in terms of hand preference strength. In particular, wild capuchins presented a weaker manual preference than captive capuchins did. This finding indicates that, despite having a preferred hand, wild capuchins were more inclined to use their nonpreferred hand to extract the kernels from nuts, while captive capuchins tended to use their preferred hand more exclusively to extract the food from the tube.

As noted by several authors, integrating results obtained in captivity with studies in natural conditions is essential for understanding the ecological validity of experimental studies (e.g., McGrew and Marchant 1997). However, one problem in trying to compare findings on handedness between captive and wild primates is the lack of common measures between the two settings (Hopkins and Cantalupo 2005; Hopkins 2013b). From this point of view, the present study has the great advantage of having identified nut kernel extraction, a foraging activity that wild bearded capuchin monkeys of FBV habitually perform in their natural environment and, from a motor point of view, mirrors the tube task, the experimental task most commonly used to measure manual preference in nonhuman primates in captivity. The two tasks share some crucial motor demands, such as using one supporting hand to hold and spatially adjust the manipulandum while the other hand performs fine movements of the digits to extract the food from inside it. Thus, the fact that capuchin monkeys in the wild presented a significant pattern of manual preference in bimanual coordination behavior during nut consumption seems to support the ecological validity of the tube task as a measure for manual preference in primates. Moreover, the difference in the strength of manual preference between monkeys in the wild and those in captivity allows us to notice the potential effects of these two different living environments on the development of motor behavior in capuchins.

Several nonmutually exclusive factors may have contributed to the fact that individuals in the wild are less lateralized than individuals living in captivity. First, perhaps the differing demands of extracting nut kernels in some way diminish the bias evident in the tube task. On the one hand, as in the tube task, independent finger movements were consistently noted in kernel extraction of the different nut species, with a predominant involvement of the distal phalanx of the index finger either alone or in conjunction with the distal phalanx of one or more additional fingers (see Supplementary Video S3). On the other hand, whereas the tube task required captive capuchins to insert one or more fingers into the tube to dig out a soft material (i.e., a slice of ripe banana) along a smooth surface, the extraction of the nut kernel attached firmly to an enclosing shell may require greater use of vigorous movements.

Another possible factor that might have increased the manual preference strength of captive capuchin monkeys tested is the novelty of the tube task. Fagot and Vauclair (1991) proposed that, in addition to the complexity of the task, the novelty of the task could also induce stronger manual preferences. Several studies in other nonhuman primate species have shown that experimental tasks presented for the first time gave rise to stronger manual preferences than spontaneous and familiar activities (e.g., Chapelain et al. 2006; Schweitzer et al. 2007; Zhao et al. 2012). Unfortunately, in these studies, the new tasks were often also rather complex from a motor point of view, making it difficult to disambiguate the relative effect of novelty and complexity. In our study, the tasks involved the same motor actions; however, while nut consumption is a spontaneous and habitual activity for wild capuchins, the tube task was a new task for the captive capuchins (Spinozzi et al. 1998). Unfortunately, the novelty of the task does not explain the more consistent use of the same hand in the action planning studies reported above, since the task was novel for both captive and wild capuchin monkeys (Sabbatini et al. 2016; Truppa et al. 2021).

Finally, living in more complex and challenging environments, such as the natural environment, might influence the development of motor behavior in capuchin monkeys, including perhaps the strength of manual laterality. This finding is consistent with recent studies on the motor planning abilities of capuchin monkeys. Capuchins in the laboratory (Sabbatini et al. 2016) and capuchins in the wild (Truppa et al. 2021) were tested with a second‐order motor planning task aimed to investigate their ability to plan their grasp on the basis of the spatial orientation of the object and the action to be performed once the object was grasped. The monkeys were presented with a horizontal dowel/stick resting on two brackets that held it in a position approximately 10 cm above the floor/ground. Food was placed on one end of the stick, alternating between the two ends across trials. The number of “comfortable” grips performed by the monkeys was evaluated. Considering that a comfortable grip is one that guarantees the shortest and most linear trajectory for bringing the end with the food to the mouth, only those grips in which the radial part of the hand was turned toward the end with the food were considered “comfortable.” The results showed that wild individuals almost always make comfortable grips, alternating the use of their two hands depending on the position of the food on the stick (right or left). In contrast, captive capuchins were less flexible in their behavior. Their greater tendency to use their preferred hand, led to a higher number of awkward grasps that they then had to adjust to bring the end with the food to their mouth (Truppa et al. 2021). Moreover, since the tasks used by Sabbatini et al. (2016) and Truppa et al. (2021) were novel to both captive and wild capuchins and were basically the same in terms of task demands, these findings are not in line with the idea that the difference in manual preference strength between the two samples may be related to the novelty of the task or different task demands.

Manual preference strength is a measure of manual laterality that has always been less studied than preference direction (right or left). However, it represents a fundamental aspect of manual laterality, which, as in the case of studies on capuchin monkeys, can reveal important information. It is therefore essential that research takes greater account of this aspect of manual laterality.

## 5 Conclusions

The results of our study confirm that natural foraging tasks requiring bimanual coordination are most likely to induce significant manual preferences at the population level. This evidence adds to numerous other studies, suggesting that the ability to use hands in a differentiated and coordinated manner may have played a key role in the evolution of manual laterality in primates by promoting the development of more specialized, lateralized systems for efficient execution. It remains to be better understood if, in this evolutionary process, some aspects of bimanual coordination may have been most relevant, including that the hands may move simultaneously (*synchrony*), perform different motor movements (*differentiation*), work in concert to achieve the same goal (*complementarity*), or make continuous adjustments during the action (*continuity*).

Our findings on capuchin monkeys provide a broader research landscape in which the study of manual functions, such as laterality, includes nonhuman primate populations in their natural environment. There is, in fact, a growing trend toward conducting studies that compare and integrate observations made in captivity with those made in the natural environment (Lopresti‐Goodman and Villatoro‐Sorto 2022) and, in some cases, observations made on natural populations living in different habitats (Falótico et al. 2022). This approach is very important for obtaining information on the long‐term effects of the various types of environmental challenges and living conditions on individuals' motor development. Furthermore, conducting similar studies in wild primate populations would be important to expand the database on laterality in natural foraging activities. As Hopkins and Meguerditchian (2025) pointed out in their review of current findings on the expression of population‐level behavioral and brain asymmetries in nonhuman primates, it is difficult to make inferences about the existence of population‐level handedness within and between species when most studies are statistically underpowered because of their small sample sizes. Overcoming this limitation is particularly challenging when studying primates in their natural habitats; therefore, it would be important to conduct similar studies in the future, which may facilitate the aggregation of data for subsequent analyses. Finally, our study highlights the importance of analyzing other measures beyond manual preference direction, such as manual preference strength. In some studies, this may also include manual performance measures, such as the speed at which each hand performs the same action (e.g., Truppa et al. 2024). Overall, a multimeasure approach to the study of manual laterality can provide a more complete picture of individuals' laterality patterns.

There is a general lack of studies evaluating bimanually differentiated behaviors, especially in natural settings, in nonhuman primates, which limits our ability to compare their manual behavior with that of humans. Relative duration/accuracy of movement, strength of different hands/limbs, and starting hand/limb have been used frequently to assess hand and forearm asymmetries in humans (see Dexheimer et al. 2024 for a review), but very rarely in nonhuman primates (e.g., Fragaszy and Mitchell 1990). Overall, studies with humans seem to support a bihemispheric model of motor control, wherein (1) distinct control strategies are employed by each hand, and (2) these strategies lead to differentiated specializations of the dominant and nondominant hands for particular movement functions. Specifically, the dominant hemisphere appears to be specialized for movement components that emphasize smoother trajectories and enhanced energetic efficiency, exemplified by tasks such as writing and throwing. In contrast, the nondominant hemisphere is likely specialized for movement aspects that provide stabilization against unpredictable forces originating from both the body and the external environment (Dexheimer et al. 2024). It remains unclear whether nonhuman primates share some or all of these features of manual control with humans.

## Author Contributions

**Valentina Truppa:** conceptualization, data curation, formal analysis, funding acquisition, investigation, methodology, project administration, resources, supervision, visualization, writing – original draft, writing – review and editing. **Ilaria Soraci:** data curation, formal analysis, visualization, writing – review and editing, conceptualization. **Luca Antonio Marino:** conceptualization, funding acquisition, investigation, visualization, resources, writing – review and editing. **Dorothy M. Fragaszy:** conceptualization, funding acquisition, project administration, resources, writing – review and editing. **Patrícia Izar:** conceptualization, funding acquisition, project administration, resources, writing – review and editing. **Elisabetta Visalberghi:** conceptualization, funding acquisition, investigation, project administration, writing – review and editing, supervision, resources.

## Supporting information


**Video S1:** Extracting behaviors during nut consumption.

**Video S2:** Other manipulative behaviors during nut consumption.

**Video S3:** Finger movements during kernel extraction of different nut species.

## Data Availability

Data that support the statistical analyses of this study are included in the article table 1. Data for comparative analysis with individuals in captivity are available in Spinozzi et al. 1998 (p. 187, Table 2, crouched condition).
